# High-performance polarization-sensitive photodetectors on two-dimensional *β*-InSe

**DOI:** 10.1093/nsr/nwab098

**Published:** 2021-05-31

**Authors:** Zhinan Guo, Rui Cao, Huide Wang, Xi Zhang, Fanxu Meng, Xue Chen, Siyan Gao, David K Sang, Thi Huong Nguyen, Anh Tuan Duong, Jinlai Zhao, Yu-Jia Zeng, Sunglae Cho, Bing Zhao, Ping-Heng Tan, Han Zhang, Dianyuan Fan

**Affiliations:** Institute of Microscale Optoelectronics, International Collaborative Laboratory of 2D Materials for Optoelectronics Science and Technology, College of Physics and Optoelectronic Engineering, Shenzhen University, Shenzhen 518060, China; Institute of Microscale Optoelectronics, International Collaborative Laboratory of 2D Materials for Optoelectronics Science and Technology, College of Physics and Optoelectronic Engineering, Shenzhen University, Shenzhen 518060, China; Institute of Microscale Optoelectronics, International Collaborative Laboratory of 2D Materials for Optoelectronics Science and Technology, College of Physics and Optoelectronic Engineering, Shenzhen University, Shenzhen 518060, China; Institute of Nanosurface Science and Engineering, Guangdong Provincial Key Laboratory of Micro/Nano Optomechatronics Engineering, Shenzhen University, Shenzhen 518060, China; Institute of Microscale Optoelectronics, International Collaborative Laboratory of 2D Materials for Optoelectronics Science and Technology, College of Physics and Optoelectronic Engineering, Shenzhen University, Shenzhen 518060, China; State Key Laboratory of Superlattices and Microstructures, Institute of Semiconductors, Chinese Academy of Sciences, Beijing 100083, China; Institute of Nanosurface Science and Engineering, Guangdong Provincial Key Laboratory of Micro/Nano Optomechatronics Engineering, Shenzhen University, Shenzhen 518060, China; Institute of Microscale Optoelectronics, International Collaborative Laboratory of 2D Materials for Optoelectronics Science and Technology, College of Physics and Optoelectronic Engineering, Shenzhen University, Shenzhen 518060, China; Department of Physics and Energy Harvest-Storage Research Center, University of Ulsan, Ulsan 680-749, South Korea; Department of Physics and Energy Harvest-Storage Research Center, University of Ulsan, Ulsan 680-749, South Korea; Institute of Microscale Optoelectronics, International Collaborative Laboratory of 2D Materials for Optoelectronics Science and Technology, College of Physics and Optoelectronic Engineering, Shenzhen University, Shenzhen 518060, China; Institute of Microscale Optoelectronics, International Collaborative Laboratory of 2D Materials for Optoelectronics Science and Technology, College of Physics and Optoelectronic Engineering, Shenzhen University, Shenzhen 518060, China; Department of Physics and Energy Harvest-Storage Research Center, University of Ulsan, Ulsan 680-749, South Korea; State Key Laboratory of Supramolecular Structure and Materials, Jilin University, Changchun 130012, China; State Key Laboratory of Superlattices and Microstructures, Institute of Semiconductors, Chinese Academy of Sciences, Beijing 100083, China; Institute of Microscale Optoelectronics, International Collaborative Laboratory of 2D Materials for Optoelectronics Science and Technology, College of Physics and Optoelectronic Engineering, Shenzhen University, Shenzhen 518060, China; Institute of Microscale Optoelectronics, International Collaborative Laboratory of 2D Materials for Optoelectronics Science and Technology, College of Physics and Optoelectronic Engineering, Shenzhen University, Shenzhen 518060, China

**Keywords:** 2D materials, polarization, photodetectors, *β*-InSe, Raman spectra

## Abstract

Two-dimensional (2D) indium selenide (InSe) has been widely studied for application in transistors and photodetectors, which benefit from its excellent optoelectronic properties. Among the three specific polytypes (*γ*-, *ϵ*- and *β*-phase) of InSe, only the crystal lattice of InSe in *β*-phase (*β*-InSe) belongs to a non-symmetry point group of $D_{6h}^4$, which indicates stronger anisotropic transport behavior and potential in the polarized photodetection of *β*-InSe-based optoelectronic devices. Therefore, we prepare the stable p-type 2D-layered *β*-InSe via temperature gradient method. The anisotropic Raman, transport and photoresponse properties of *β*-InSe have been experimentally and theoretically proven, showing that the *β*-InSe-based device has a ratio of 3.76 for the maximum to minimum dark current at two orthogonal orientations and a high photocurrent anisotropic ratio of 0.70 at 1 V bias voltage, respectively. The appealing anisotropic properties demonstrated in this work clearly identify *β*-InSe as a competitive candidate for filter-free polarization-sensitive photodetectors.

## Teaser

p-type *β*-InSe has been successfully prepared, and its high anisotropic electronic and optoelectronic properties have been demonstrated by angle-resolved polarized Raman and applied on high-performance polarization-sensitive photodetectors.

## INTRODUCTION

With regard to extracting the polarization information of incident light, polarization-sensitive photodetectors (PSPDs) exhibit significant practical application in both military and civil areas, like bio-imaging [1], remote sensing [2], night vision [3] and helmet-mounted sight for fighter planes [4]. Optical filters combined with polarizers are usually needed for traditional photodetectors to realize polarized light detection. But this increases the size and complexity of devices [5]. To obtain a small-sized PSPD, one-dimensional (1D) nanomaterials with geometrical anisotropy, such as nanowires, nanoribbons and nanotubes [6,7], have been used as sensitive materials for PSPDs, which can directly identify the polarization information of incident light without any optical filters and polarizers. However, it is not an easy task to pattern and integrate these 1D nanochannels for mass production of PSPDs [3,8–10].

Atomically layered two-dimensional (2D) semiconductors with low crystal symmetry have shown great potential in micro-nano PSPDs recently due to their intrinsically in-plane anisotropic properties [11,12]. For example, SnS, ReS_2_, GeS_2_, GeAs_2_, AsP and black phosphorus (BP) exhibit an obvious in-plane anisotropy behavior in carrier transport, thermal conductivity, electrical conductivity, thermoelectric transport and optical absorption processes. They have potential application in PSPDs, polarization ultrafast lasers, polarization field effect transistors and polarization sensors [9–15]. Among them, BP-based PSPDs have the highest photocurrent anisotropy ratio of 0.59 [16], benefitting from its high carrier mobility and the strong in-plane anisotropy coming from the low-symmetry puckered honeycomb crystal structure [17,18]. But with BP-based optoelectronic devices it is hard to get rid of the ambient degradation problem [19,20]. 2D layered indium selenide (InSe), which also has high carrier mobility and is more stable than BP in an atmospheric environment [21], exhibits huge potential for application in high-performance optoelectronic and electronic devices [22–27]. In addition, the anisotropic optical and electronic properties of 2D layered InSe have already been demonstrated in 2019 [28,29]. It is worth noting that InSe crystal has three specific polytypes, which are in *β*, *γ* and *ϵ* phases, respectively [30]. Among them, InSe in *γ*-phase and *ϵ*-phase belongs to the $C_{3V}^5$ and $D_{3h}^1$ symmetry groups respectively. Only the InSe in *β*-phase (*β*-InSe) belongs to the non-symmetry point group of $D_{6h}^4$, indicating that *β*-InSe exhibits better anisotropic optoelectronic properties than the other two polytypes.

In this work, p-type *β*-InSe single crystals are successfully prepared by controlling the composition via temperature gradient method. The morphology, structure and stability of the *β*-InSe flake are systematically investigated and characterized. The anisotropic nature of *β*-InSe has been unveiled by angle-resolved polarized Raman spectroscopy. Then experiments and theoretical calculations are carried out to explore the optical and electrical anisotropy of *β*-InSe. Finally, the polarization-sensitive photoresponse of photodetectors based on the *β*-InSe is demonstrated. The results reveal that a high photocurrent anisotropy ratio among single 2D-material-based PSPDs of 0.70 could be obtained from the *β*-InSe-based PSPDs, indicating its great potential in high-performance micro-nano PSPDs.

## RESULTS

**Figure 1.**
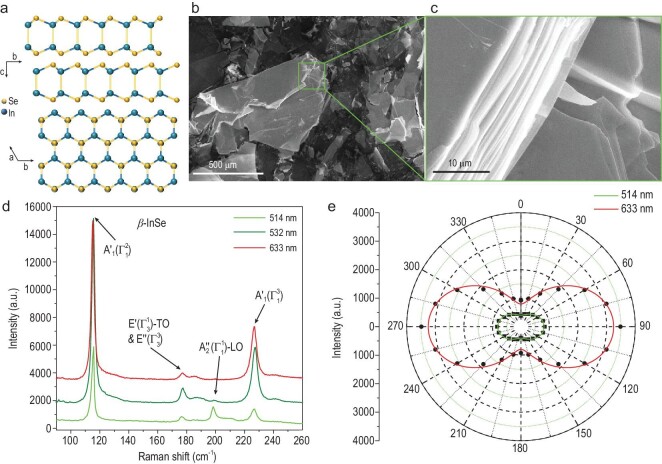
Structure and characterization of the few-layer *β*-InSe flake. (a) Front and top views of the hexagonal structure of the *β*-InSe crystal. (b and c) The SEM images of the same *β*-InSe flake with different magnification, revealing the layered structure of the *β*-InSe flake. (d) Raman spectra of *β*-InSe under 514, 532 and 633 nm laser excitations. (e) Polar plot of the angle-resolved polarized Raman intensity of ${\rm{A}}_1^{\rm{^{\prime}}}( {{\rm{\Gamma }}_1^3} )$ vibrational modes of *β*-InSe under 514 and 633 nm excitations.

The single crystal of *β*-InSe in this work is grown via a temperature gradient method (see the Methods section), which has a hexagonal crystal structure stacked in AB order belonging to the non-symmetric $D_{6h}^4$ space group as shown in Fig. 1a. The morphology of the InSe samples is investigated by scanning electron microscopy (SEM) (Fig. 1b and c) and the cross section of the samples reveals the obvious layered structure of InSe. According to the results of the energy-dispersive x-ray spectroscopy (EDS), shown in Fig. S1 in the online supplementary file, the atomic ratio of indium to selenium is closed to 1 : 1 as designed, with a little excess of selenium. A photo of InSe bulk and the corresponding X-ray diffraction (XRD) pattern are shown in Fig. S2, where the diffraction peaks are in agreement with the standard crystal data of *β*-phase InSe (JCPDS No. 34-1431). The lattice parameters have been calculated to be a = 4.001 Å and c = 16.618 Å based on the XRD measurement. Figure 1d shows the unpolarized Raman spectra of the *β*-InSe flake on a silicon (Si) wafer, which has been calibrated by standard Si peak at 520.7 cm^–1^. Three typical InSe Raman peaks at 115, 176 and 227 cm^–1^ corresponding to the out-of-plane ${\rm{A}}_1^{\rm{^{\prime}}}( {{\rm{\Gamma }}_1^2} )$, in-plane ${\rm{E^{\prime}}}( {{\rm{\Gamma }}_3^1} ) - {\rm{TO}}\ {\&} \ {\rm{E^{\prime\prime}}}( {{\rm{\Gamma }}_3^3} )$ and out-of-plane ${\rm{A}}_1^{\rm{^{\prime}}}( {{\rm{\Gamma }}_1^3} )$ vibrational modes of InSe can all be observed under 514, 532 and 633 nm excitations. One resonance Raman peak at 199 cm^–1^ corresponding to the out-of-plane ${\rm{A}}_2^{{\rm{^{\prime\prime}}}}$(${\rm{\Gamma }}_1^1$)-LO vibrational mode can be observed under 514 nm (2.41 eV) excitation [31]. Figure 1e shows the polar plot of the angle-resolved polarized Raman intensity of the ${\rm{A}}_1^{\rm{^{\prime}}}( {{\rm{\Gamma }}_1^3} )$ vibrational mode. The intensity of ${\rm{A}}_1^{\rm{^{\prime}}}( {{\rm{\Gamma }}_1^3} )$ vibrational mode changes periodically with the polarization angle of the excitation, indicating a strong optical anisotropy of *β*-InSe. The same tendency of the polarized Raman spectra could be observed under resonance excitation (514 nm, Fig. S3) and non-resonance excitation (633 nm, Fig. S4). The angle-resolved polarized Raman spectra of *β*-InSe can be understood by the classical Raman selection rules and the crystal structure [10,32]. As shown in Fig. S5, when the thickness of the *β*-InSe sheet decreases, the anisotropic ratio of the ${\rm{A}}_1^{\rm{^{\prime}}}( {{\rm{\Gamma }}_1^3} )$ mode increases, which indicates that a thin layer possesses relatively high anisotropic Raman properties. The Raman spectroscopy of *β*-InSe has demonstrated a high in-plane optical anisotropy, indicating a great opportunity to exploit *β*-InSe-based PSPDs.

**Figure 2.**
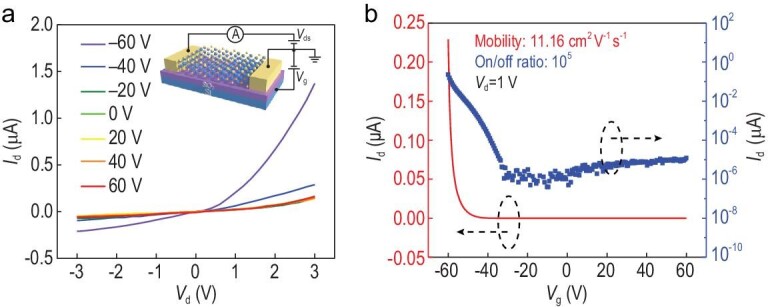
Electrical characterization of a few-layer *β*-InSe FET on a SiO_2_/Si substrate. (a and b) Output transfer curves obtained from an 8-nm-thick device on a silicon substrate with 300 nm SiO_2_ at room temperature and in the air. The inset of (a) is the schematic structure of a few-layer *β*-InSe FET. For the transfer curves in (b), the drain-source voltage is 1 V. The channel length and width of the device are 5 μm and 5 μm, respectively.

The back-gated structure of the *β*-InSe-based field effect transistor (FET) is shown in the inset of Fig. 2a. The thickness of the *β*-InSe flake is ∼8 nm (Fig. S6), determined by atom force microscopy (AFM). The gate tunable output characteristics (*I*_ds_ − *V*_ds_) (Fig. 2a) of the *β*-InSe-based FET show that the source-drain current *I*_ds_ increases at the same bias (*V*_ds_) when gate voltage (*V*_g_) varies from +60 V to −60 V. As shown in Fig. 2b, the transfer characteristics (*I*_ds_ − *V*_g_) at *V*_ds_ = 1 V of the FET exhibit a p-type dominated ambipolar conduction behavior with an on/off current modulation of 10^5^, which is one order larger than the requirement (10^4^) for complementary metal oxide semiconductor (CMOS) logic devices [14]. The p-type conduction behavior is attributed to the existence of indium vacancies during the crystal growth. The atomic ratio of In to Se is ∼44 : 56 according to the results of EDS spectra in Fig. S1b and the as-generated indium vacancies can serve as acceptor dopants [33,34]. The calculated field effect electron mobility is ∼11.16 cm^2^ V^–1^ s^–1^ at room temperature in ambient air. This relatively low mobility might result from the scattering effect of air and SiO_2_ substrate [14]. In addition, the *β*-InSe flakes and their FET exhibit good stability in air due to its specific ABAB stacking mode (as shown in Fig. S7), and there is almost no change on the morphology (Fig. S8) and Raman spectra (Fig. S9) of the *β*-InSe flake as well as the *I*_ds_ − *V*_g_ curves of *β*-InSe FET (Fig. S10a) after being exposed in the air for 10 days. Besides, *I*_ds_ − *V*_ds_ curves change little after being tested 13 times with a 1-minute interval (Fig. S10b), indicating a good operation stability.

**Figure 3.**
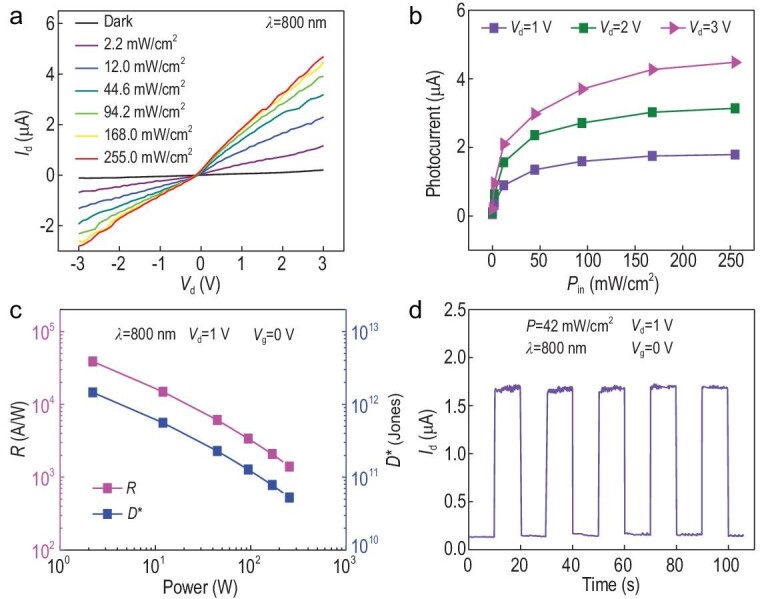
Photodetection performance of a few-layer *β*-InSe photodetector (L = 10 μm, W = 10 μm). (a) Output curves of a few-layer *β*-InSe photodetector in dark and under illumination with different excitation intensities at $\lambda$ = 800 nm, *V*_g_ = 0 V. (b) Power-dependent photocurrent of the layer *β*-InSe photodetector at *V*_ds_ = 1 V, *V*_ds_ = 2 V and *V*_ds_ = 3 V, respectively. (c) Responsivity and specific detectivity as a function of illumination intensity at $\lambda$ = 800 nm, *V*_ds_ = 1 V, *V*_g_ = 0 V. (d) A test of the photoswitching stability for the *β*-InSe device acquired at $\lambda$ = 800 nm and P = 42 mW cm^–2^, *V*_ds_ = 1 V, *V*_g_ = 0 V, showing that the rise time is ∼22 ms and the decay time is ∼24 ms.

For the photoresponse performance, as shown in Fig. 3a and b, *I*_ds_ and the photocurrent (*I*_ph_) at *V*_g_ = 0 V of the *β*-InSe-based FET increase as the power intensity (*P*_in_) of the laser beam is enlarged ($\lambda$ = 800 nm) and reaches saturation when *P*_in_ is higher than 255.0 mW cm^–2^. As the *P*_in_ dependent responsivity results show in Fig. 3c, the responsivity reaches the highest value of 194 A W^–1^ at the intensity of 2.2 mW cm^–2^ and 1 V bias. It is noteworthy that the responsivity is inversely proportional to the *P*_in_ of the laser beam, which results from the trap-related photogating effect reported previously [22,35,36]. The specific detectivity (D^*^) reaches the highest value of 1.45 × 10^12^ Jones at the intensity of 2.2 mW cm^–2^ and 1 V bias. The deduction can be indirectly proven by the *P*_in_ dependence of *I*_ph_ at different bias in Fig. 3b, which satisfies the power law relation of *I*_ph_ ∝ *P*^α^_in_. The simulated index of power law α are ∼0.26 (at *V*_ds_ = 1 V), ∼0.26 (at *V*_ds_ = 2 V) and ∼0.28 (at *V*_ds_ = 3 V), which are all much lower than 1, indicating a strong trap-related carrier recombination in the *β*-InSe-based FET [37]. For the dynamic photoresponse of the device, *I*_ds_ can be reproducibly switched from the ‘on’ state to the ‘off’ state with the rise time of ∼620 μs and the decay time of ∼540 μs, which indicates good reproducibility of the device, as shown in Fig. 3d and Fig. S11. The relatively slow response time may be improved by using graphene electrodes that have been proven in recent reports [38–44].

## DISCUSSION

**Figure 4.**
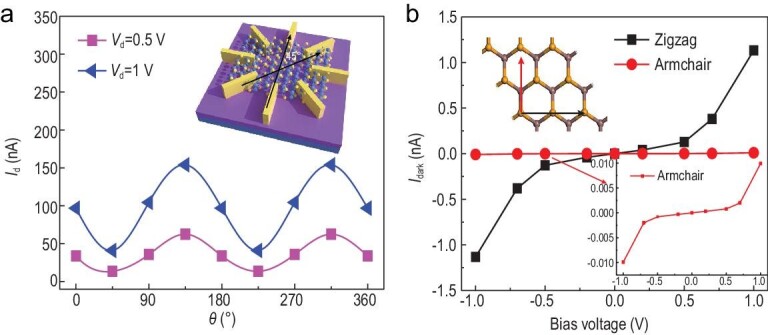
Anisotropic transport properties of few-layer *β*-InSe. (a) Angular dependence of the source-drain current of the device at *V*_ds_ = 0.5 V and *V*_ds_ = 1 V, respectively. The inset of (a) is the structure of the device for the angle-dependent transport behavior determination. (b) The DFT calculated dark current versus bias voltages along the zigzag and armchair directions. The top-left inset is the top view of the *β*-InSe structure, and the bottom-right inset is the dark current along the armchair direction.

The *β*-InSe-based FET with eight electrodes (inset of Fig. 4a) has been fabricated to determine the angle-dependent transport behavior of *β*-InSe. The thickness of the *β*-InSe flake is measured to be 6.8 nm by AFM (Fig. S12). As shown in Fig. 4a, the angle-dependent dark current of device has the ratios of 4.62 (*V*_ds_ = 0.5 V) and 3.76 (*V*_ds_ = 1 V) for the maximum to minimum dark current at two orthogonal orientations. It suggests the obvious in-plane anisotropic conduction of *β*-InSe. Quantum transport calculation based on non-equilibrium Green's function density functional theory (NEGF-DFT) calculation has been carried out to determine the origin of the distinct anisotropic conduction behavior of *β*-InSe. As shown in Fig. S13, the supercell of the *β*-InSe hexagonal lattice has two perpendicular directions, armchair and zigzag. Due to the electrostatic potential profile difference between two directions, and the fact that the lattice energy-barrier scattering to Bloch waves along the armchair direction is more significant (Fig. S14), a large anisotropy of dark current can be predicted (Fig. 4b); the dark current along the zigzag direction might be theoretically two orders greater than that along the armchair direction.

**Figure 5.**
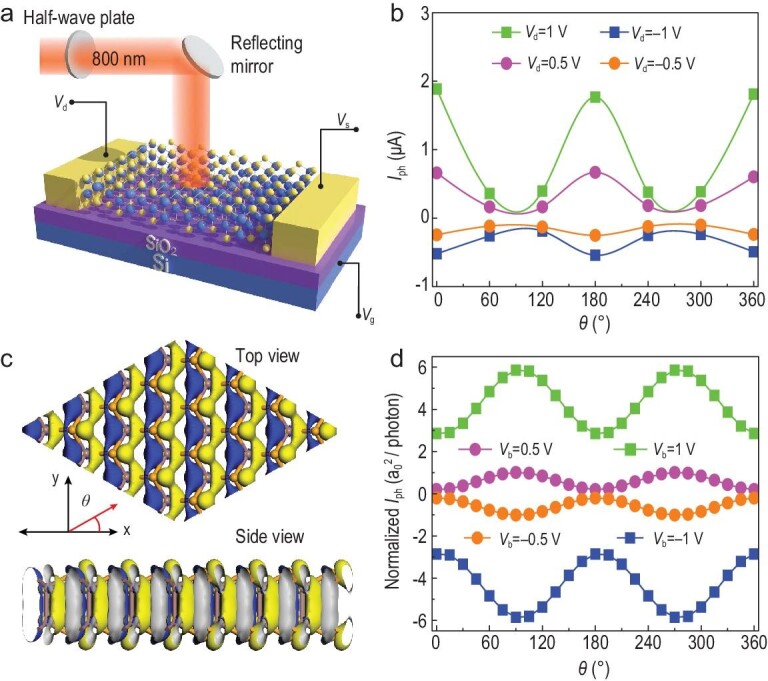
Polarization-sensitive photoresponse of the *β*-InSe FET. (a) The configuration for angle-dependent transport behavior determination. (b) Angular dependence of the photocurrent of the device at *V*_ds_ = 0.5 V and *V*_ds_ = 1 V, respectively. (c) Top view and side view of the contributing orbital to the photocurrent. Red arrow indicates the light polarization angle *θ*. (d) The quantum transport calculation of the photocurrent with different polarized angle *θ* for ±0.5 V and ±1.0 V bias voltages, when the light of the wavelength of 800 nm is irradiated.

Then the configuration in Fig. 5a is set up for testing the polarization sensitivity of *β*-InSe FET. As shown in Fig. 5b, the photocurrents dependent on the polarization angle demonstrate a shape of cos*θ* function in a period of π. In addition, the measured ratio of maximum to minimum photocurrent is ∼5.66 at *V*_ds_ = 1 V. As a result, the photocurrent anisotropy ratio of our *β*-InSe devices is ∼0.70 according to the formula (*I*_phmax_ − *I*_phmin_)/(*I*_phmax_ + *I*_phmin_), which is ranking high among the single 2D-material-based PSPDs as shown in Table 1, except for one Te-nanosheet-based polarized infrared imaging system, which has an anisotropy ratio of 0.8 for 2.3 μm light [45]. The photocurrent changing with the polarized angle *θ* of incident light at 800 nm (1.55 eV) along the armchair direction has also been simulated by DFT. From the band structure of the *β*-InSe (Fig. S15), an indirect bandgap ∼1.2 eV is observed. Figure 5c illustrates the orbital isosurface contributing to the photocurrent. Arrows indicate the light polarization angle *θ*, which is with respect to x axis along the armchair direction. The electron cloud isosurface shows that the main contribution is along the Se 4*p_y_* direction. The transition possibility increases when the polarization direction parallels with the orbital dipoles. Hence, as shown in Fig. 5d, when θ = 0° and 180°, light polarization is perpendicular to the Se 4*p_y_*and the photocurrent reaches the minimum. When θ = 90°, light polarization is in line with Se 4*p_y_* and the photocurrent reaches the maximum. A 0.67 anisotropic ratio of the photoresponse can be extracted from the theoretical calculation, which is a little smaller than the experimental one, 0.70. The reason for this smaller calculated one is that we did not take other electrons into account for the anisotropic photoresponse besides the Se 4*p_y_*. Both the experimental and theoretical results demonstrate a significant in-plane anisotropic photoresponse of *β*-InSe that is crucial for the realization of filter-free micro-nano PSPDs. As shown in Fig. S16, when the thickness of the *β*-InSe sheet decreases, the anisotropic ratio of the photoresponse performance will increase, which is in accordance with the layer-dependent anisotropic Raman result.

**Table 1.** Anisotropic performance comparison of photodetectors made with different materials.

PSPDs	Bias (V)	Wavelength (nm)	Anisotropic ratio
			
ReS_2_ [9]	1	532	0.54
SnS-SnS_x_Se_(1−x)_ [13]	7	532	0.082
ReSe_2_ [14]	1	633	0.50
GeSe [3]	2	808	0.36
GeAs_2_ [15]	1	532	0.33
GeS_2_ [10]	10	325	0.35
GeSe/MoS_2_ [1]	0	532	0.35
BP [16]	0.15	1550	0.59
Te [45]	1	2300	0.8
BP/InSe [8]	0	633	0.83
BP on WSe_2_ [46]	0.5	1550	0.71
TiS_3_/Si [47]	−0.5/0	660	0.29/0.44
Bi_2_Te_3_ [48]	1	635	0.23
Bi_2_Te_3_/CuPc [49]	0.01	650	0.44
InSe (this work)	1	800	0.70

## CONCLUSION

In summary, a p-type *β*-InSe single crystal is successfully prepared via temperature gradient method, which can be exfoliated into 2D layered flakes by tape-assisted mechanical exfoliation. The anisotropic nature of the *β*-InSe has been revealed by angle-resolved Raman. The out-of-plane vibrational modes exhibit pronounced periodic variations with the polarization angle of the excitations. Besides, a good stability of *β*-InSe flakes and their FET devices has been proven by AFM measurement and multi-repeat electrical performance test. The theoretical calculations are in good agreement with the experimental results in that there is strong anisotropic transport and a polarization-sensitive photoresponse in 2D layered *β*-InSe flakes. The photocurrent anisotropic ratio of the *β*-InSe photodetector reaches 0.70, which is ranking high among the single 2D-material-based PSPDs. The strong anisotropic Raman, transport and photoresponse properties of the *β*-InSe enable its great application potential in filter-free PSPDs.

## METHODS

### Preparation of ***β***-InSe single crystal

A temperature gradient method has been used for *β*-InSe single crystal synthesis. A mixture of In and Se powders at a molar ratio 1 : 1 was placed in double-wall cleaned quartz ampoule down to a residual pressure of ∼10^–^^4^ Torr. The ampoules were kept in a vertical electrical furnace. Then the ampoules were slowly heated to 723 K at a rate of 20 K-1h to let indium and selenium react. After the reaction, the ampoules were heated to 1023 K at a rate of 10 K-1h and maintained at this temperature for 16 h to insure a complete reaction throughout the whole specimen. They were cooled down to 723 K slowly at a rate of 1 K-1h and finally cooled down at a rate of 20 K-1h to room temperature. The single crystal obtained was 25 mm in length and 13 mm in diameter. Increasing the soaking time to give enough time for a complete reaction between raw materials may be one way to improve the quality of the sample. And the rate of cool down of the furnace could be optimized, for example, 0.5 K-1h instead of 1 K-1h.

### Device fabrication and measurement

Polarized Raman spectra have been obtained by a Jobin Yvon/HORIBA LabRam ARAMIS Raman spectrometer with 514, 633 and 785 nm lasers, where the polarization of the laser has been tuned by a Spectra-Physics automatic polarizer. The *β*-InSe FETs were fabricated according to the following steps. The few-layer *β*-InSe flakes were obtained by typical scotch tape assisted mechanical exfoliation of bulk *β*-InSe and transferred to a Si/SiO_2_ (300 nm) wafer by using a polydimethylsiloxane (PDMS) thin film. Then methyl methacrylate (MMA) and polymethyl methacrylate (PMMA) have been spin-coated on the surface of the Si/SiO_2_ wafer step-by-step. After that, electron-beam lithography (Raith Pioneer Two) and electron-beam evaporation were carried out to pattern the micro-scale Cr/Au (5 nm/50 nm) electrodes on *β*-InSe flakes. Following a lift-off process, *β*-InSe FETs were obtained. The *β*-InSe FETs were evaluated on a home-built probe station with a Keithley 4200 Semiconductor Parameter Analyzer, which is open to the air. The laser used for the photoconductivity measurements was generated from a coherent femtosecond laser.

### Calculation method

We use a method based on NEGF-DFT to calculate the linear polarized photocurrent of *β*-InSe. *β*-InSe bulk structure belongs to P63/_mmc_ space group. To calculate the photocurrent flow through *β*-InSe under linearly polarized light, we construct a two-probe device model for the armchair and zigzag directions. Generalized gradient approximation (GGA) expressed by the Perdew-Burke-Ernzerhof (PBE) is used to describe the exchange correlation functional. The maximum force acting on each atom becomes smaller than 0.03 eV/Å. The energy is optimized until it changes less than 10^–^^5^ eV/atom per step. The criteria for stress and displacement convergence are 0.05 GPa and 0.001 Å, respectively.

## Supplementary Material

nwab098_Supplemental_File
