# Supplementary material for: High-performance polarization-sensitive photodetectors on two-dimensional β-InSe
Source: Natl Sci Rev. 2021 May 31;9(5):nwab098. doi: 10.1093/nsr/nwab098 (PMC9113105; doi:10.1093/nsr/nwab098)
Supplement: nwab098_Supplemental_File [file nwab098_supplemental_file.docx]

Supplementary Information for *National Science Review*

**High performance polarization sensitive photodetectors on two dimensional** ***β*-InSe**

Zhinan Guo^1^, Rui Cao^1^, Huide Wang^1^, Xi Zhang^2^, Fanxu Meng^1^, Xue Chen^5^, Siyan Gao^2^, David K. Sang^1^, Thi Huong Nguyen^3^, Anh Tuan Duong^3^, Jinlai Zhao^1^, Yu-Jia Zeng^1^, Sunglae Cho^3^, Bing Zhao^4^, Ping-Heng Tan^5^, Han Zhang^1,*^, and Dianyuan Fan^1^

^1^ Institute of Microscale Optoelectronics, International Collaborative Laboratory of 2D Materials for Optoelectronics Science and Technology, College of Physics and Optoelectronic Engineering, Shenzhen University, Shenzhen, 518060, P. R. China.

^2^ Institute of Nanosurface Science and Engineering, Guangdong Provincial Key Laboratory of Micro/Nano Optomechatronics Engineering, Shenzhen University, Shenzhen 518060, P. R. China.

^3^ Department of Physics and Energy Harvest-Storage Research Center, University of Ulsan, Ulsan 680-749, Republic of Korea.

^4^ State Key Laboratory of Supramolecular Structure and Materials, Jilin University, Changchun 130012, China.

^5^ State Key Laboratory of Superlattices and Microstructures, Institute of Semiconductors, Chinese Academy of Sciences, Beijing 100083, China.

* Correspondence and requests for materials should be addressed to Han Zhang (email: hzhang@szu.edu.cn).

Zhinan Guo, Rui Cao, Huide Wang, and Xi Zhang contribute equally to this work.


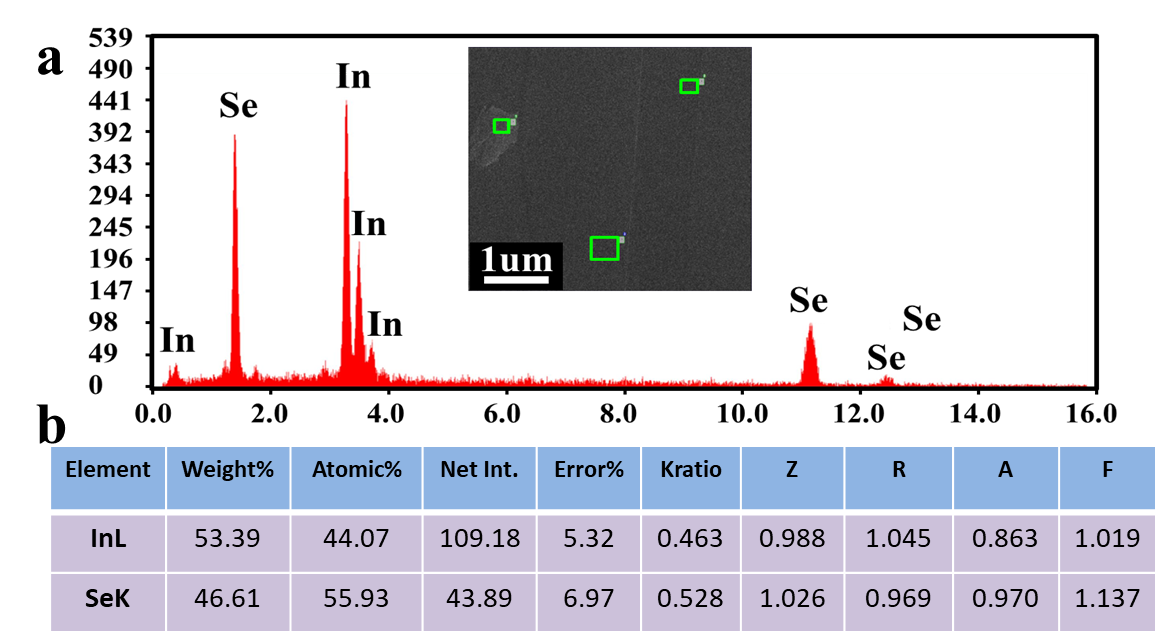


**Figure S1.** EDS test of the *β*-InSe sheet. (a) EDS spectra and (b) data of the *β*-InSe sheet for the scanned area of the green box in the inset, showing the atomic ratio of In atomic and Se atomic of the *β*-InSe sheet is almost 1:1.

Inductively coupled plasma atomic emission spectroscopy (ICP-AES, Agilent 7700x) has used to obtain a more accurate atomic ratio of In to Se. The mass percentage results of the dissolved *β*-InSe sample output by the ICP-AES measurement are In 560.839 ppb for In and 410.602 ppb for Se, which means the real atomic ratio of In to Se is 48.35:51.65.


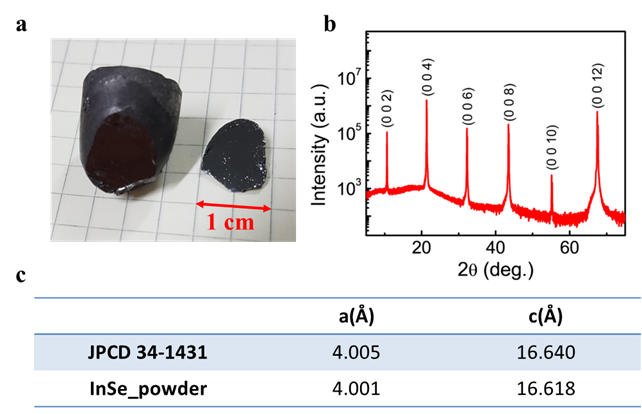


**Figure S2.** (a) Photo and (b,c) XRD result of InSe sample, which demonstrate the material is *β*-InSe.


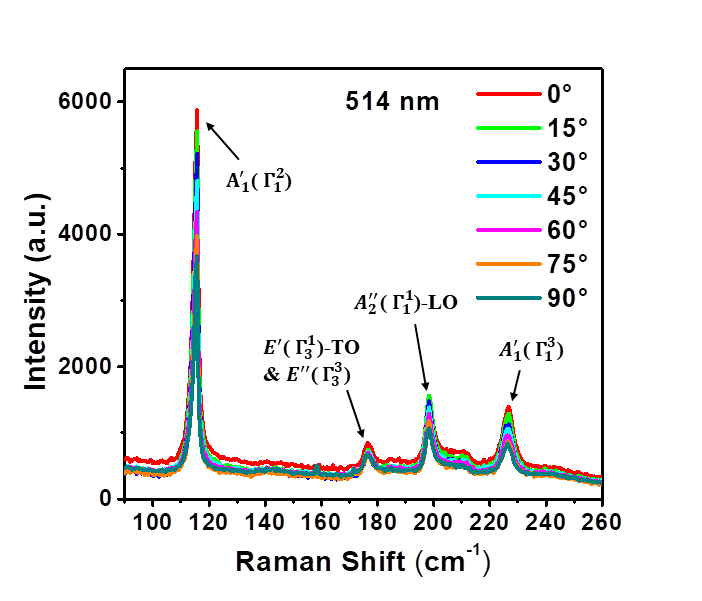


**Figure S3.** Polarized Raman spectra of the *β*-InSe sheet with different polarization angle under 514 nm excitation.


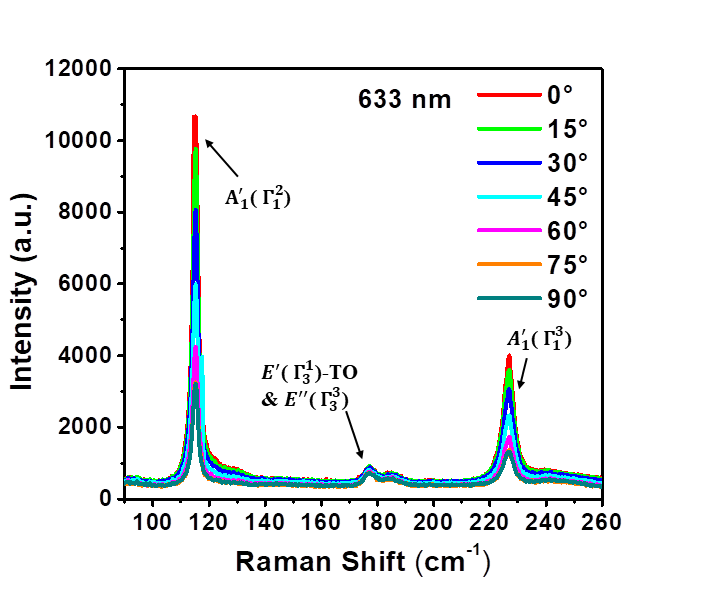


**Figure S4.** Polarized Raman spectra of the *β*-InSe sheet with different polarization angle under 633 nm excitation.


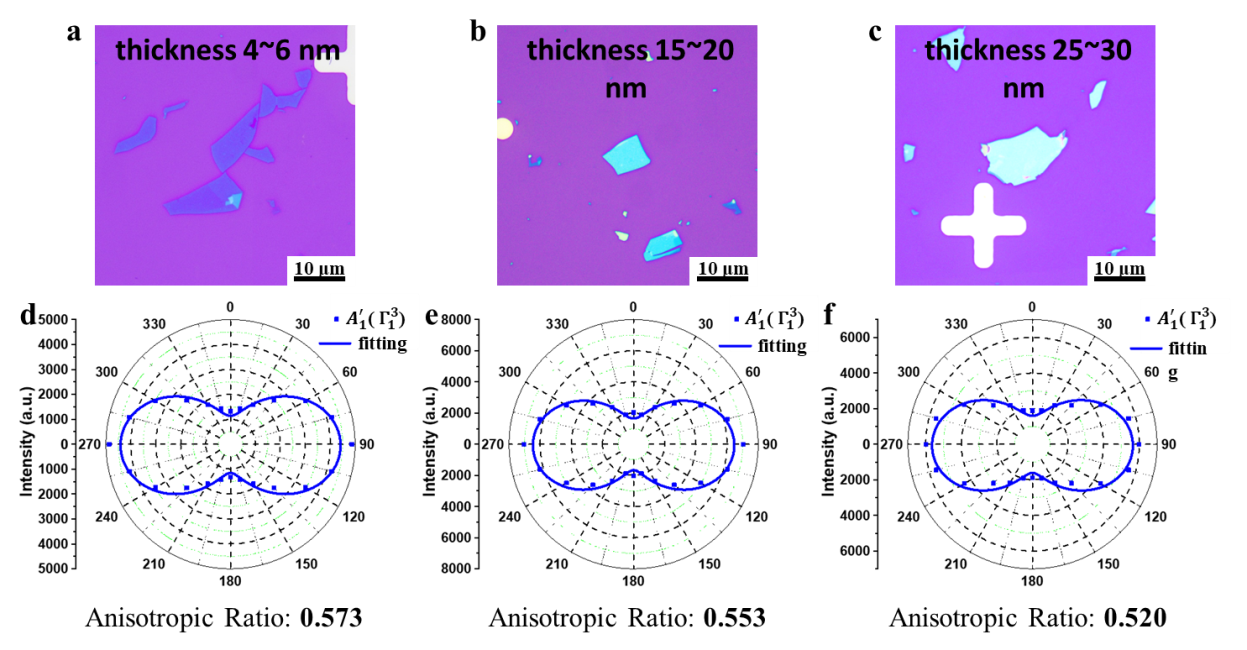


**Figure S5.** Optical images and thickness-dependent polarization-Raman spectra of three *β*-InSe nanosheets with different thickness: thin one (a, d), middle one (b, e), and thick one (c, f).


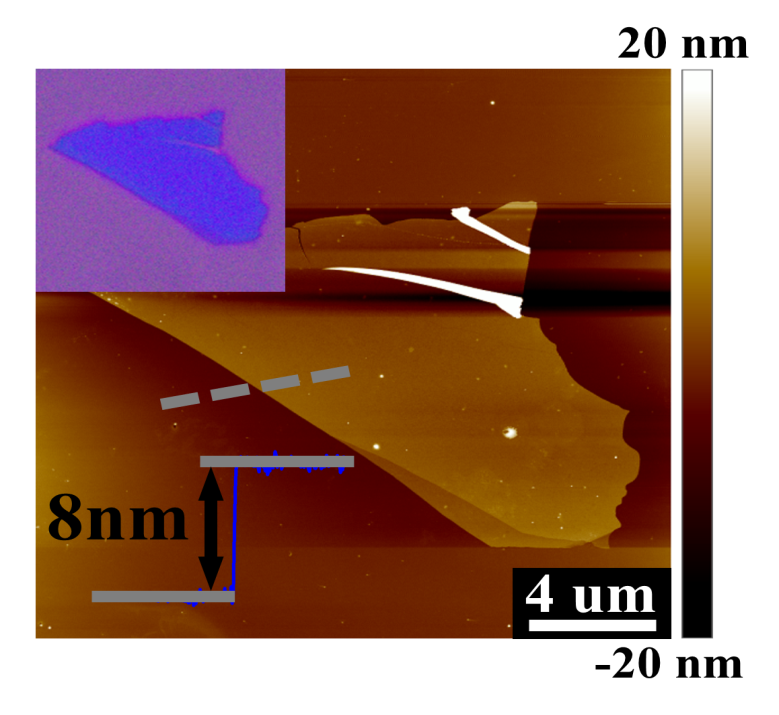


**Figure S6.** The optical (inset) and AFM images of the few-layer *β*-InSe sheet for FET fabrication.


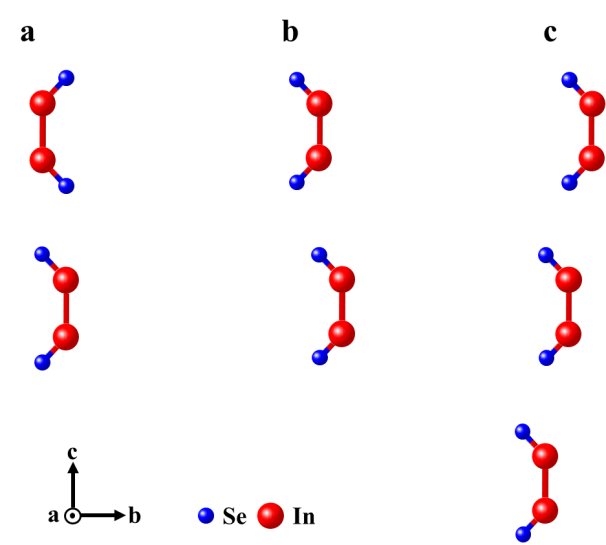


**Figure S7.** Unit cell of InSe phases: (a) *β*-InSe, (b) *ε*-InSe and (c) *γ*-InSe.


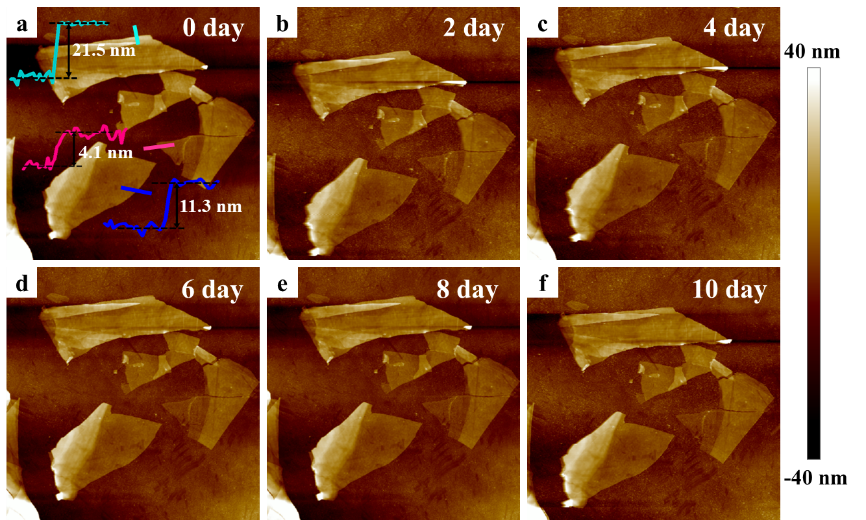


**Figure S8.** Morphology of few-layer *β*-InSe sheets at different time in the air. AFM images of a few-layer *β*-InSe sheet (~ 4.1 nm, ~ 11.3 nm, and ~ 21.5 nm) exposed to air for (a) 0 day, (b) 2 days, (c) 4 days, (d) 6 days, (e) 8 days (f) 10 days, revealling the robust stability of the few-layer *β*-InSe sheet with different thickness.


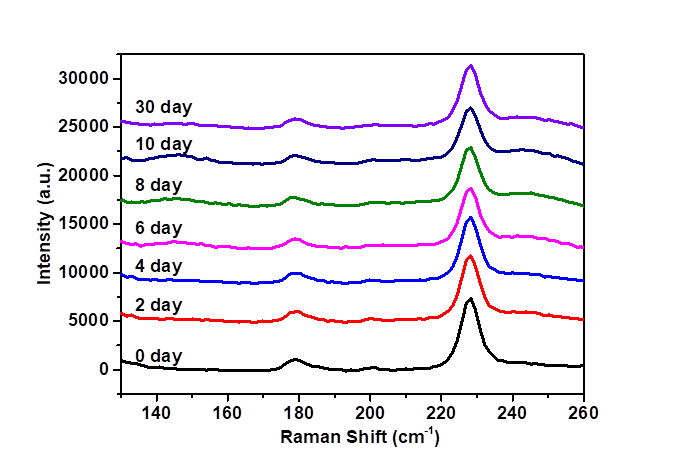


**Figure S9.** Raman spectra of few-layer *β*-InSe sheet at different time in the air.


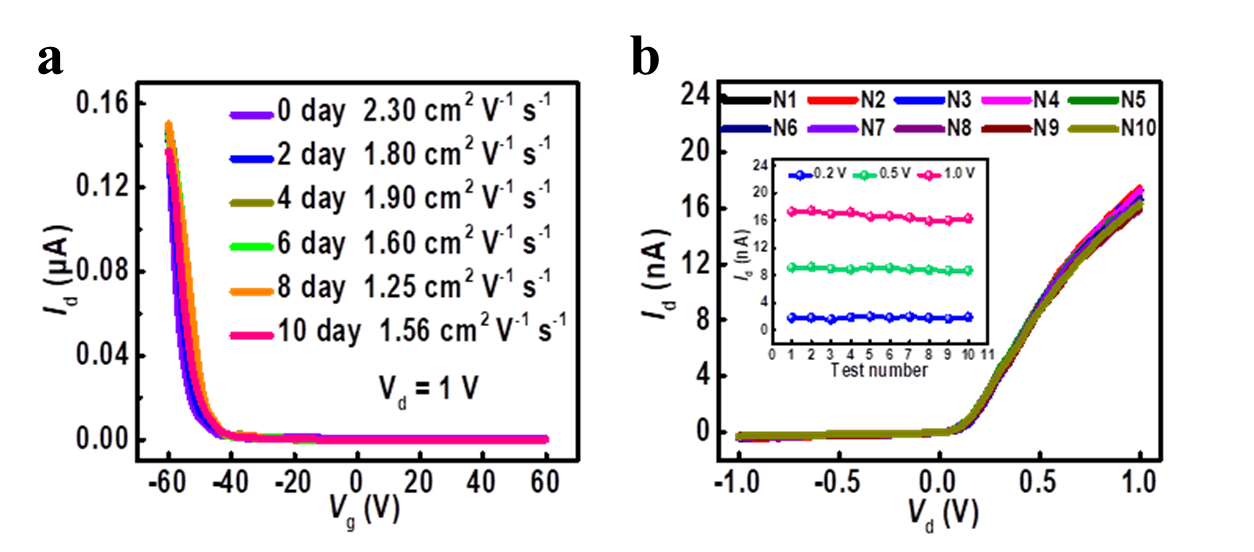


**Figure S10.** Stability of electrical characterization of a few-layer *β*-InSe FET. (a) Current to gate voltage curve obtained from a *β*-InSe FET device after air exposure for 0-10 day for comparison at *V*_ds_ = 1 V. (b) Current to drain-source voltage curve obtained from a *β*-InSe FET device at thirteen times with 1-minute interval. Inset: Current to test number curve at *V*_ds_ = 1 V, *V*_ds_ = 2 V and *V*_ds_ = 3 V, respectively.


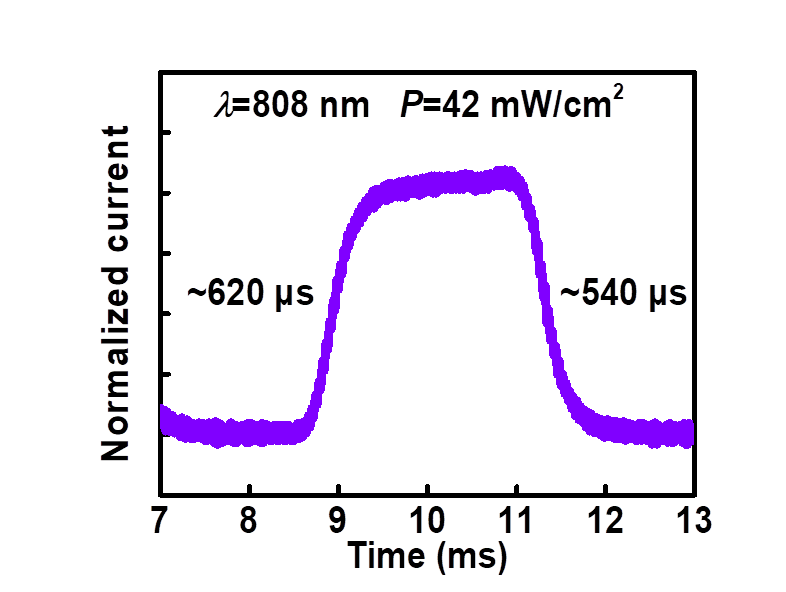


**Figure S11.** Response time of the device under 808 nm light illumination during one cycle.

**The calculation process of the performance parameters of the photodetector**

The responsivity (*R*) can be calculated by the formula:

*R* =*I*_ph_ / (*P*_in_ *A*)

where *I*_ph_ is the photocurrent, *P*_in_ is the laser power density, *A* is area of the photodetector channel.

The specific detectivity (*D**) can be calculated by the approximate calculation formula:

*D** =*R A*^1/2^ / (2e *I*_dark_ )^1/2^

where e is the electron charge, and *I*_dark_ is the dark current [1].

**Reference**

[1] Wu F. *et al*. AsP/InSe Van der Waals Tunneling Heterojunctions with Ultrahigh Reverse Rectification Ratio and High Photosensitivity. *Adv Funct Mater* 2019; **29**: 1900314.


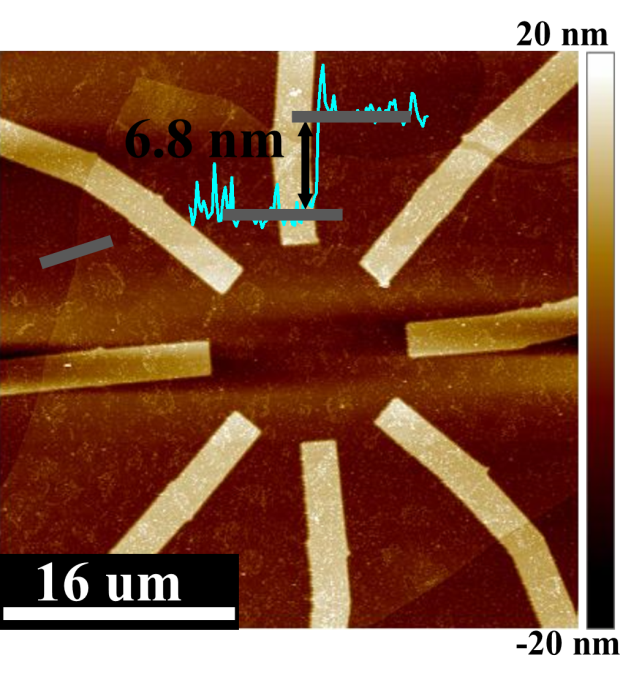


**Figure S12.** Morphology of the *β*-InSe based device for determining the angle-dependent transport behavior and the thickness of *β*-InSe flake is ∼6.8 nm according to the profile.

**Principle investigation of strong anisotropy of photo-response in** ***β*-InSe via density functional theory**

1. **Construction of structure**

*β*-InSe bulk structure belongs to P63/_mmc_ space group. The optimized bulk *β*-InSe has lattice constants *a1* = *a2* = 4.113 Å. As shown in the Figure 1aa, the supercell of *β*-InSe hexagonal lattice has two perpendicular directions, armchair and zigzag. To calculate the photocurrent flow through *β*-InSe under linearly polarized light, we construct a two-probe device model for the armchair and zigzag directions. As shown in the Figure S5, the top and side views of the *β*-InSe photodetector in the armchair and zigzag direction.


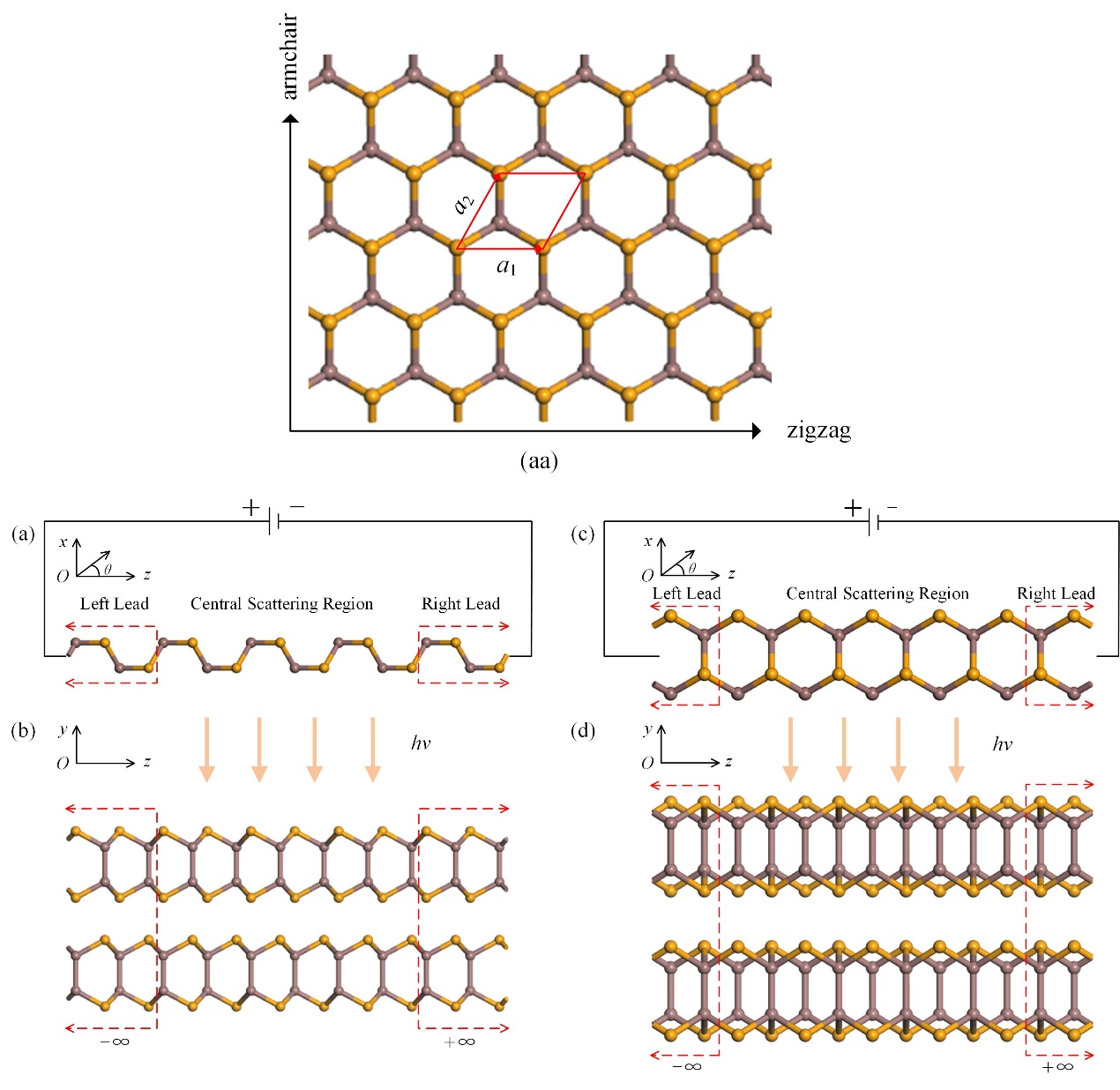


**Figure S13**. Construction of the *β*-InSe crystal structure. (aa) Top-view of *β*-InSe supercell. *a_1_* and *a_2_* denotes the basis vectors of the hexagonal lattice. Arrows indicate the armchair and zigzag directions. (a) Top-view and (b) side-view two-probe device along armchair direction. (c) Top-view and (d) side-view two-probe device along zigzag direction. The angle *θ* denotes the polarization angle of linear light with respect to the current direction. The red dashed lines represent the left and right lead, respectively. The remaining is the scattering region. The arrows indicate the polarized light used to irradiate the entire scattering region.

The two-probe device consists of three parts: a scattering region, the left and right lead structures, which extend to ±∞ along the transport direction, respectively. The current direction is set along the armchair direction in (a)-(b) and zigzag direction in (c) and (d) of the *β*-InSe. Bias was applied on the left lead against the right lead. The central section is where the photocurrent flows from the left lead to right lead acrossing the scattering region. The whole scattering region is irradiated perpendicularly with linearly polarized light. The polarization direction forms an angle θ with respect to the current direction.


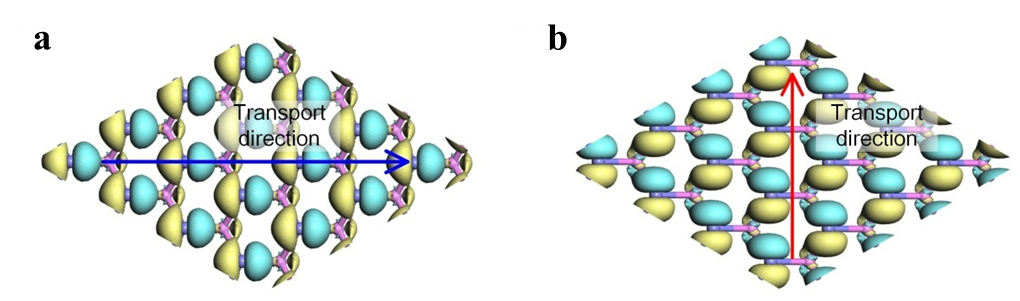


**Figure S14.** The calculated Bloch waves of Se orbitals. (a) Bloch waves of Se 4*px* orbital along the armchair direction; (b) Bloch waves of Se 4*py* orbital along the zigzag direction.


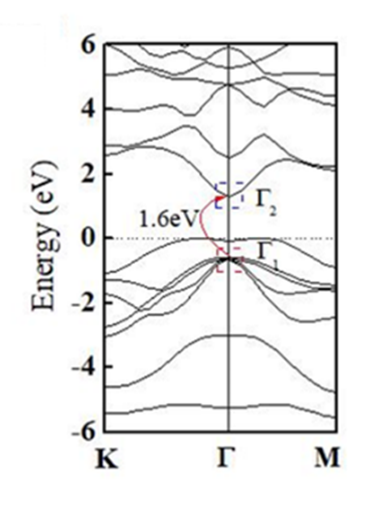


**Figure S15.** The band structure of *β*-InSe.

From the band structure of the *β*-InSe, an indirect bandgap about 1.2 eV is observed. The valence band maximum (VBM) occurs between the K point and Γ point, while the conduction band minimum (CBM) is located at the Γ point. Fig. S15 shows the composition of the transition molecular orbitals of Г_1_ and Г_2_ which are two superposition orbitals mainly denoted by Se 4p_y_ and 4p_x_ atomic orbitals.

1. **Computational details**

Here, structural optimization is performed using the Nanodcal. Generalized gradient approximation (GGA) expressed by the Perdew-Burke-Ernzerhof (PBE) is used to describe the exchange correlation functional. The maximum force acting on each atom becomes smaller than 0.03 eV/Å. The energy is optimized until it changes less than 10^-5^ eV/atom. The criterion for stress and displacement convergence are 0.05 GPa and 0.001 Å, respectively. In addition, the double-ξ plus polarization (DZP) basis set is employed in this calculation. To avoid the interaction between slabs in the neighboring unit cells, a vacuum space along the z direction is set to 20 Å.

1. **Photocurrent calculations**

In this work, we use a method based on density functional theory within Keldysh nonequilibrium Green’s function (NEGF-DFT) to calculate the photocurrent. Specifically, the electron-photon interaction is treated as a perturbation on the self-consistent Hamiltonian of the system (*H_0_*). Therefore, the total Hamiltonian of the electron-photon system can be written as:

$$\hat{H}=\hat{H}_{0}+\frac{e}{m_{0}}\mathbf{A}\cdot\hat{\mathbf{p}}$$

Where **A** is the polarization vector of the light. The Green’s functions are written as :

$$G^{<}=G^{0r}(i\Gamma_{L}f_{L}+i\Gamma_{R}f_{R}+\sum_{ph}^{<})G^{0a}$$

$$G^{>}=G^{0r}(i\Gamma_{L}(1-f_{L})+i\Gamma_{R}(1-f_{R})+\sum_{ph}^{>})G^{0a}$$

Where *Γ* is the linewidth function of the electrodes, represents the coupling of the device scattering region to the electrodes. *G^0r^* is the retarded Green’s functions without photon. *G^0a^* is the advanced Green’s functions without photon. The photoinduced current is calculated by as follow:

Where *α* represent the lead of source/drain. *T_α_*(*E*) is the effective transmission coefficient of lead α, which can be expressed as:

$$T_{\alpha}(E)=Tr\left\{i\Gamma_{\alpha}[(1-f_{\alpha})G_{ph}^{<}+f_{\alpha}G_{ph}^{>}]\right\}$$

Where *Γ_α_* represents the line-width function which describes the interactions between the lead *α* and the scattering regions. *f_α_* is the Fermi distribution function of lead *α*. *G_ph_* ^<^and *G _ph_* ^>^ denote the zeroth order approximations of the smaller and greater Green’s functions based on the Keldysh equation. From above, we calculate the normalized photocurrent, the photoresponse function can be written as :

$$R=\frac{I_{ph}}{eI_{\omega}}$$

Where *I_ω_* is the photon flux.

**Thickness dependent anisotropy photoresponse of the *β*-InSe photodetector**


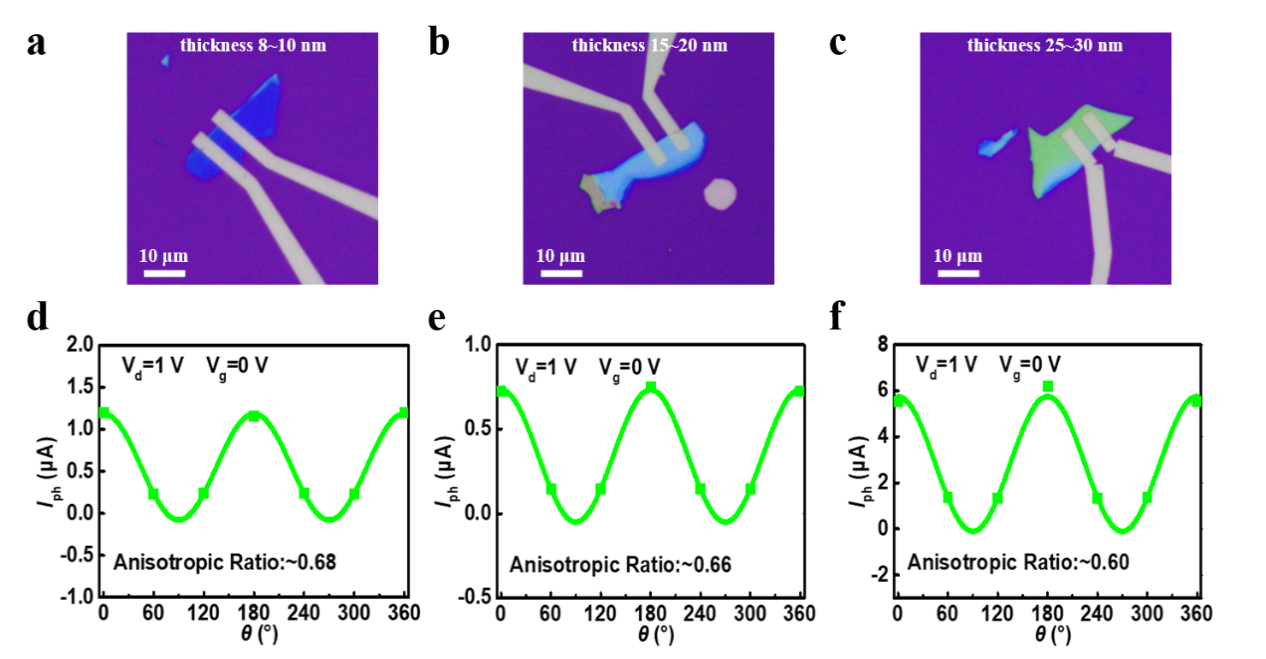


**Figure S16.** Optical images and thickness-dependent anisotropy photoresponse of the *β*-InSe photodetectors: thin one (a, d), middle one (b, e), and thick one (c, f).
